# A case report of esophageal cancer in a patient with pemphigus vulgaris: A coincidence or something beyond that?

**DOI:** 10.1002/cnr2.1896

**Published:** 2023-09-14

**Authors:** Kamran Balighi, Zeinab Aryanian, Shadi Balighi, Ifa Etesami, Parvaneh Hatami

**Affiliations:** ^1^ Autoimmune Bullous Diseases Research Center Tehran University of Medical Sciences Tehran Iran; ^2^ Department of Dermatology, School of Medicine Razi Hospital Tehran University of Medical Sciences Tehran Iran; ^3^ Department of Dermatology Babol University of Medical Sciences Babol Iran; ^4^ School of Allied Medical Sciences Tehran University of Medical Sciences Tehran Iran

**Keywords:** case report, neoplasm, pemphigus, pemphigus vulgaris, rituximab, squamous cell carcinoma

## Abstract

**Background:**

Pemphigus is a group of rare but serious autoimmune blistering disorders, affecting skin and mucus membrane. Different reports have been published in respect to the coexistence of pemphigus with neoplasms, especially lympho‐proliferative ones.

**Case:**

Here, we have reported a patient previously diagnosed with pemphigus vulgaris (PV) who developed esophageal squamous cell carcinoma (SCC).

**Conclusion:**

Dyspepsia and dysphagia in patients with PV might not be merely due to pemphigus erosions or simply an adverse effect of systemic corticosteroid such as irritant or candidal esophagitis and should raise the suspicion of more serious conditions in case of resistant symptoms without appropriate response to treatment.

## 1 INTRODUCTION

Pemphigus is a group of serious blistering disorders characterized by the development of flaccid and painful blisters on the skin and/or mucous membranes. It is caused by producing autoantibodies against desmogleins (Dsg), which plays an important role in the maintenance of cell‐to‐cell adhesion.^1^, ^2^ Different reports have been published in respect to the coexistence of pemphigus with neoplasms, especially lympho‐proliferative ones.^3^ Here, we have reported a patient previously diagnosed with pemphigus vulgaris who developed esophageal squamous cell carcinoma. In this case, we tried to speculate regarding the exact type of association of esophageal squamous cell carcinoma and pemphigus. Different aspects of this association were also dealt with.

## 2 CASE PRESENTATION

**FIGURE 1:**
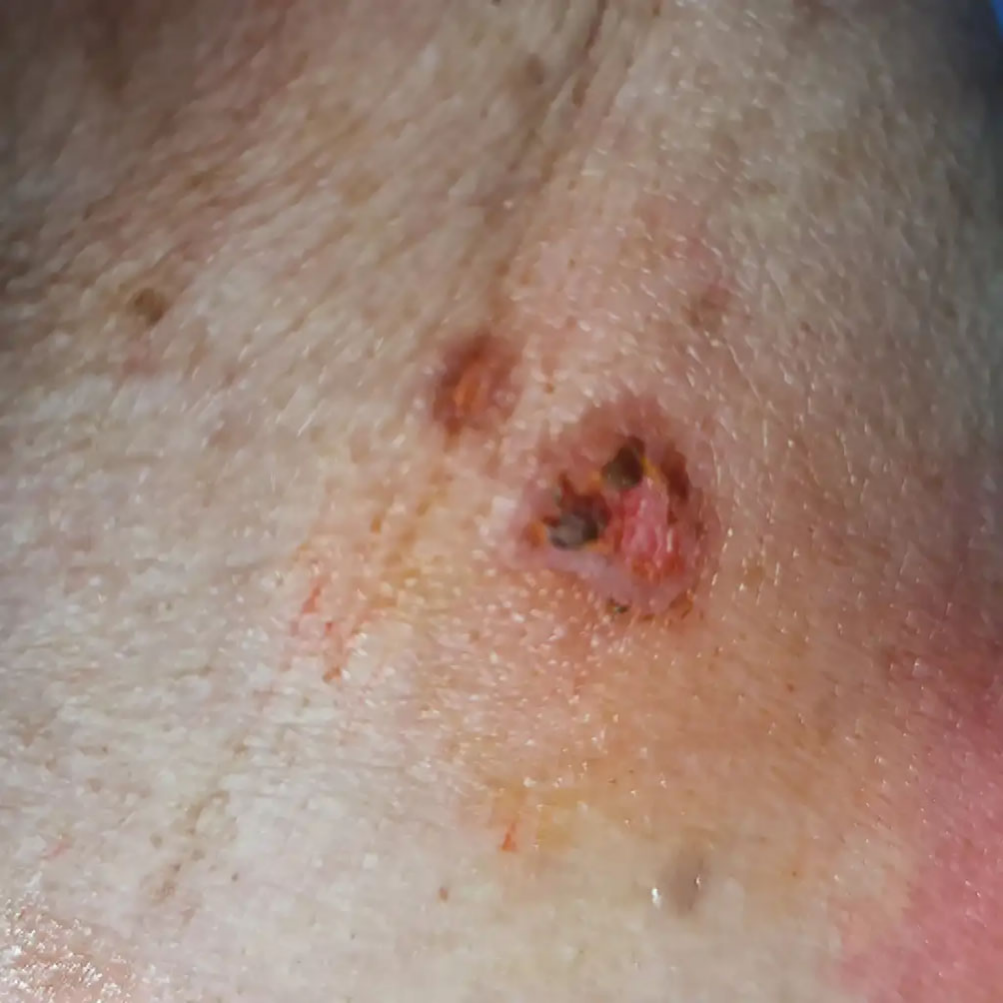
A well‐defined erythematous erosion compatible with the diagnosis of PV.

**FIGURE 2:**
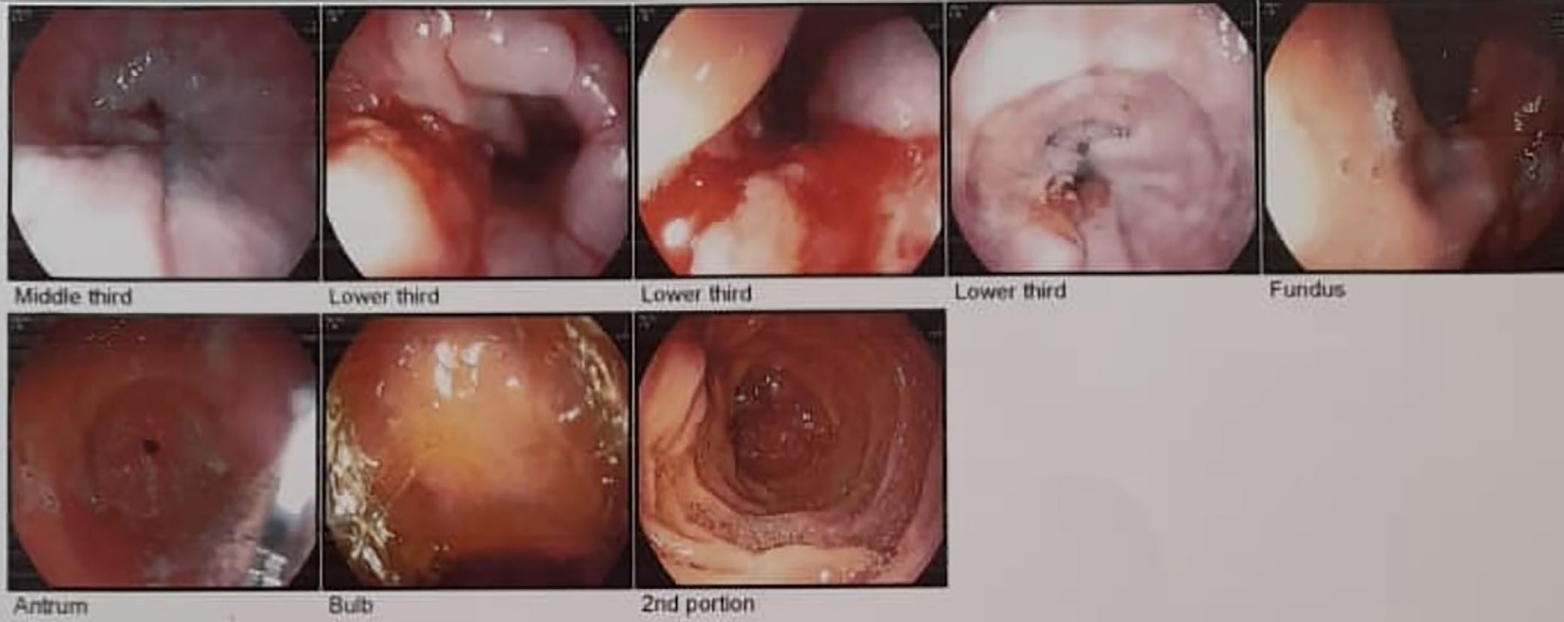
Esophageal endoscopy. Ulcerative lesion of distal esophagus.

A 61‐year‐old man with confirmed diagnosis of PV came to us at Razi Hospital, affiliated to Tehran University of Medical Sciences on March 2022, with complain of dysphagia from 2 months ago. He was firstly presented with painful oral ulcers and widespread cutaneous erosions mainly over truncal area (Figure 1). Histopathologic and direct immunofluorescence (DIF) examinations established the diagnosis of PV. His disease had initially been controlled with 80 mg/day prednisolone which tapered to 15 mg/day during 3 month and rituximab (one cycle consisting of one infusion of 500 mg every week for 4 weeks) was added to his therapeutic regimen leading to a considerable clinical improvement. After 6 months, he experienced a relapse which was controlled with 20 mg/day prednisolone. During the tapering phase, he noticed a constant dyspepsia and subsequently, underwent upper gastrointestinal (GI) endoscopy and biopsy in his city of residence which revealed a mild chronic gastritis and ulceration with moderate dysplasia at the lower part of esophagus, probably due to inflammation. After consultation with GI department, a prompt anti acid protocol besides rapid tapering of oral corticosteroid was recommended. However, dysphagia was added to patient's previous symptoms which led us to consider a more serious condition. Hence, the patient was referred to the GI department, where another endoscopic biopsy was conducted which was suggesting of invasive squamous cell carcinoma (Figure 2).

**FIGURE 3:**
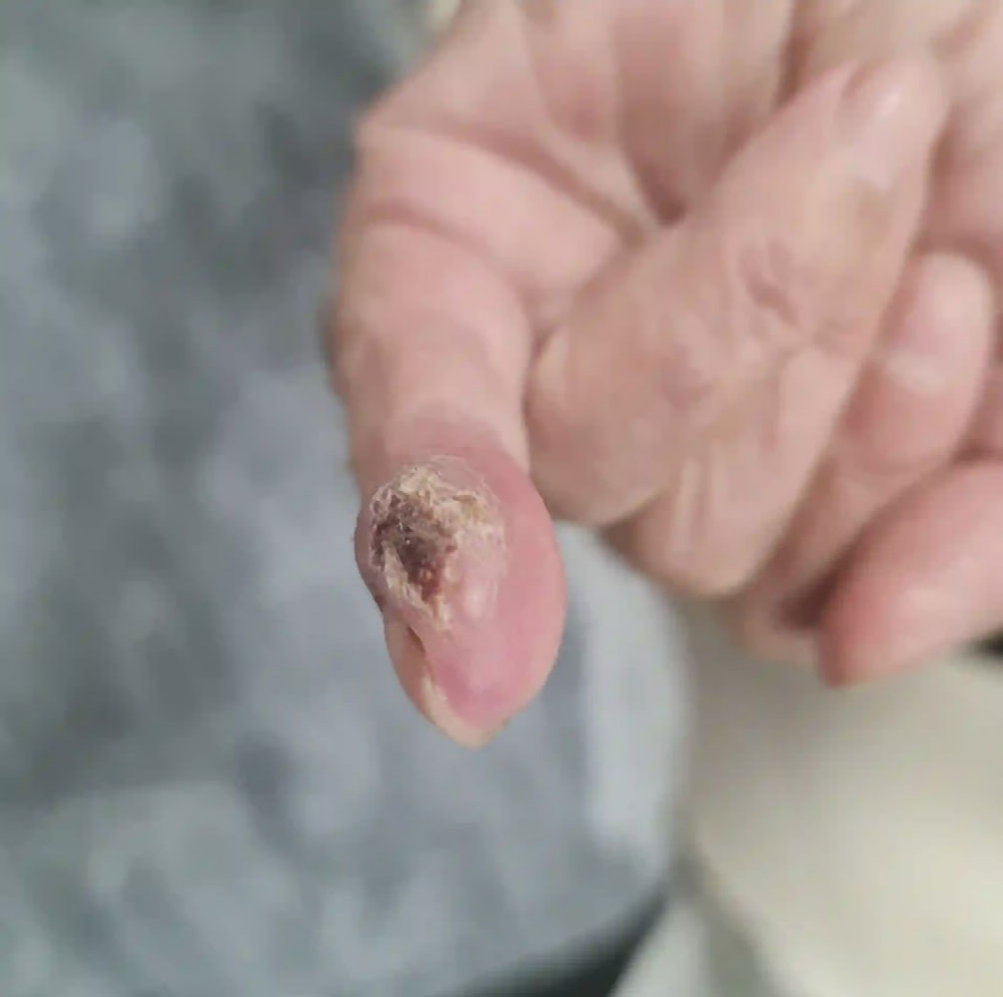
Metastatic SCC appeared during chemotherapy on patient's fingertip.

**FIGURE 4:**
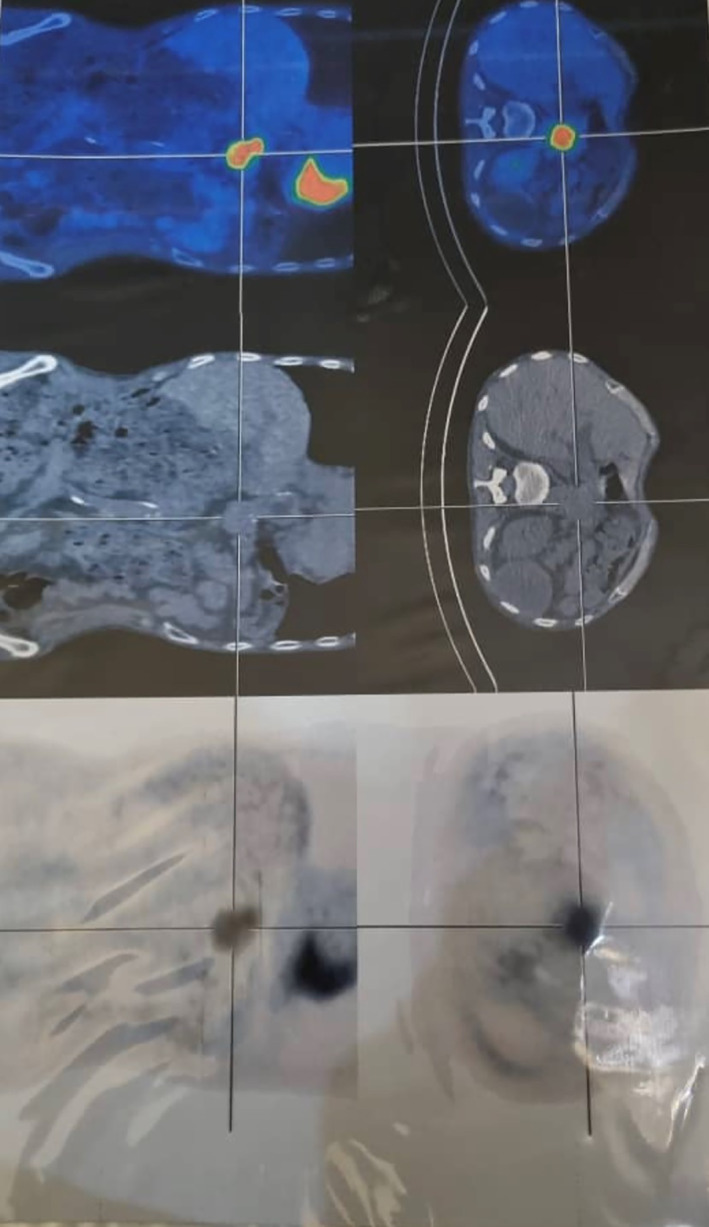
PET scan shows a large lymph node in the paraesophageal region measuring 38 × 28 mm with hypermetabolism.

After consultation with oncology department, initiation of radiochemotherapy and subsequent surgical excision was planned for patient. Before finishing of radiochemotherapy sessions, an ulcerative lesion was appeared on the tip of second finger of his right hand (Figure 3) which turned out to be a squamous cell carcinoma. Positron‐emission tomography (PET) scan also showed some para aortic lymphadenopathy (Figure 4). Considering metastatic lesion of finger and PET scan findings regarding progression of the disease, the chemotherapy regimen was changed to a more aggressive regimen and surgical amputation of digital distal phalanx was scheduled. From the dermatologic point of view, the patient has been under strict follow up and any relapse of PV has not been noted until now (5 months after amputation).

## 3 DISCUSSION

The association of pemphigus, mainly paraneoplastic pemphigus (PNP) and neoplasm has been previously reported and seems to be related to the dysregulation of immune responses.^3^ Although the neoplasm is usually detected before the onset of PNP, pemphigus can be manifested before the detection of an occult tumor in 30% of cases.^4^, ^5^ PNP following gastric cancers (gastric lymphoma and adenocarcinoma) is rarely reported.^6^, ^7^ It is worthy to note that PNP might share clinical, histopathological and serological features with PV.^8^ Hence, we could not rule out the diagnosis of PNP for our case, though his clinical and laboratory features were in favor of the diagnosis of PV. However, based on the first endoscopic examination of our case, ulceration and moderate dysplasia (rather than obvious squamous cell carcinoma) was noted when patient already had the clinical manifestation of pemphigus which has risen several questions: Was that a pemphigus erosion which dysplasia was superimposed on it or it developed on an intact mucosa? Are neoplasms at very early stages (such as moderate dysplasia) able to alter humoral or/and cellular arms of immune system to develop PNP? Is the stage of neoplasm (early‐stage tumor versus well‐formed one) important to the amount of immune system dysregulation and thus, the severity of clinical manifestation? If yes, one can infer that early‐stage neoplasm of our case led to milder mucocutaneous lesions mimicking PV, compared to those expected in PNP.

PNP is not the only cancer‐associated subtype of pemphigus. A recent study on 164 patients with pemphigus vulgaris, 19 were diagnosed with malignancy, both before or after the diagnosis of PV and the prevalence of malignancies in patients with PV was significantly higher than normal population.^9^ Warshavsky et al. concluded that PV, itself can lead to a higher rate of both solid and hematological cancers in patients suffering from PV.^9^ However, Desrosiers et al. described two cases with co‐existence of non‐melanoma skin cancer and PV and postulated that the presence of PV lesions could be merely due to koebner phenomenon.^10^

Moreover, there are some limited reports of neoplasm development following rituximab therapy^11^ which might be explained by the fact that rituximab affects both the humoral and cellular arms of the immune system.^12^ This risk might be even more prominent in patients with former dysplasia in context of treatment with immunosuppressive agents such as rituximab or even corticosteroids.

Therefore, it is not easy to speculate regarding the exact type of association of esophageal squamous cell carcinoma and pemphigus in this case.

Another important learning point from this case is the fact that dyspepsia and dysphagia in patients with PV might not be merely due to pemphigus erosions or simply an adverse effect of systemic corticosteroid such as irritant or candidal esophagitis and should raise the suspicion of more serious conditions in case of resistant symptoms without appropriate response to treatment.

Recently, we have reported an interesting case of PV who developed kaposi's sarcoma (KS) after receiving immunosuppressive agents which was initially localized to the oral cavity with features mimicking exacerbation of his pemphigus to emphasize the fact that careful examination and clinical suspicion are the key factors for correct diagnosis.^13^

Future studies are needed to shed light on this issue by further assessment of risk of developing tumors following rituximab therapy as well as the potential of developing PNP following mucosal dysplasia. Moreover, it is important to find a solution to distinguish atypical forms of PNP from PV, because early diagnosis of PNP may lead to a better prognosis for patient due to early diagnosis and even treatment of the underlying neoplastic diseases.^14^, ^15^

## AUTHOR CONTRIBUTIONS

**Kamran Balighi:** Conceptualization (equal); supervision (equal). **Zeinab Aryanian:** Formal analysis (equal); writing – original draft (equal). **Shadi Balighi:** Investigation (equal). **Ifa Etesami:** Investigation (equal); validation (equal). **Parvaneh Hatami:** Investigation (equal); writing – review and editing (equal).

## CONFLICT OF INTEREST STATEMENT

The authors have stated explicitly that there are no conflicts of interest in connection with this article.

## ETHICS STATEMENT

Ethical approval from the Medical Ethics Committee of Tehran University of Medical Sciences was provided. Written informed consent was obtained from the patient for publication of this case report and any accompanying images. A copy of the written consent is available for review by the Editor‐in‐Chief of this journal.

## Data Availability

The data that support the findings of this study are available from the corresponding author upon reasonable request.
